# An alternative, low-dissolution counter electrode to prevent deceptive enhancement of HER overpotential

**DOI:** 10.1038/s41598-022-13385-w

**Published:** 2022-06-07

**Authors:** Menna M. Hasan, Nageh K. Allam

**Affiliations:** Energy Materials Laboratory, School of Sciences & Engineering, The American University in Cairo, Cairo, 11835 Egypt

**Keywords:** Chemistry, Energy science and technology

## Abstract

Electrochemical hydrogen evolution reaction (HER) is typically studied in three-electrode system. In this system, several counter electrodes are commonly used to ensure fast kinetics, including Pt, gold, and glassy carbon. However, the extensive application of such electrodes has raised caveats on the contribution of the redox-active species dissolving from such electrodes and redepositing on the surface of the working electrode to the measured overpotential. Consequently, this has been frequently confused with the actual electrochemical signature of the working electrode catalyst, resulting in a deceptive enhancement in the recorded overpotential. This issue becomes more critical when the electrolysis measurements involve an activation step, necessitating the need for alternative counter electrodes that are stable, especially in acidic medium, which is commonly used as the electrolyte in HER studies. Herein, while we systematically unveil such problems, an alternative counter electrode that overcomes those problems is demonstrated. Specifically, the correlation between the working electrode area to that of the counter electrode, the dissolution rate of the counter electrode, and the potential range used in the activation/cleaning of the surface on accelerating the dissolution rate is explored and discussed in detail. Finally, commercial Ti mesh is demonstrated as an alternative emerging counter electrode, which is proven to be very stable and convenient to study the HER in acidic media.

## Introduction

During the past few decades, hydrogen has been introduced as a promising alternative to traditional fossil fuels. In this regard, electrolysis is the commonly used process to produce hydrogen in large quantities. In this system, Pt is the most widely used electrocatalyst for the water reduction half reaction due to its efficient electrical conductivity, high mechanical strength, and superior catalytic activity^1^. However, its use in large scale production has been greatly restricted due to its high cost. Therefore, there is a growing need to develop earth-abundant hydrogen evolution reaction electrocatalysts that possess comparable catalytic activity to that of Pt while being inexpensive. For instance, previous studies introduced molybdenum carbide combined with rGO, revealing almost the same activity towards hydrogen evolution reaction (HER) of Pt/C^2^. Also, Ni − C-based catalysts showed comparable performance to that of Pt in HER^3^. Nevertheless, almost half of the published studies related to HER used Pt as the counter electrode with no ion-exchange membrane used^4,5^. However, Pt is not stable and undergoes chemical/electrochemical dissolution during potential cycling^4,6,7^. Then, the dissolved Pt ions redeposit on the surface of the working electrode, resulting in deceptive enhancement in the measured overpotential^4,6,7^. Furthermore, even when an ion exchange membrane (Nafion) is used, Pt dissolution and deposition on the working electrode still takes place^8^. These concerns necessitated the search for alternative counter electrodes. Consequently, carbon-based materials such as glassy carbon or graphite rod have been used instead. However, the question is still there; are those electrodes stable? and if not, is there a way out?

To address this issue, herein, we investigated the stability of the most commonly used counter electrodes (Pt, Au, and glassy carbon) during the HER in acidic media. The effect of the relative area of the working electrode to the counter electrode and the potential range used in the activation/cleaning of the surface on the rate of the counter electrode dissolution and its correlation to the recorded overpotentials is fully addressed and discussed. Finally, we demonstrate the potential of commercial titanium mesh as a stable and functional counter electrode for HER in acidic media.

## Experimental methods

All the electrochemical measurements were conducted in 0.5 M H_2_SO_4_ electrolyte in a three-electrode system. For the working electrode, a very thin layer of boron carbon nitride-copper composite was deposited on graphite sheet. Hg/HgSO_4_ was used as the reference electrode while Pt foil, gold coil, glassy carbon rod, and titanium mesh were used as the counter electrode (CE). All voltage values were converted into the reversible hydrogen electrode (RHE), where E_RHE_ = E_Hg/HgSO4_ + 0.64 V + 0.059 pH. The morphology of the fabricated nanofibers was characterized with Zeiss SEM Ultra 60 field emission scanning electron microscope (FESEM). Energy Dispersive X-ray (EDX; Oxford ISIS 310, England) spectroscopy attached to the FESEM microscope was used for the elemental analysis and mapping of the fabricated electrodes, and the crystal structure using X-ray photoelectron spectroscopy (XPS, Thermo-Scientific) measurements were conducted in UHV chamber equipped with hemispherical energy analyzer (SPHERA U7) with Al Kα monochromator X-ray source (1486.6 eV), operated at Constant Analyzer Energy (CAE 50) mode.

## Results and discussion

### Effect of the activation range on CE dissolution

**Figure 1:**
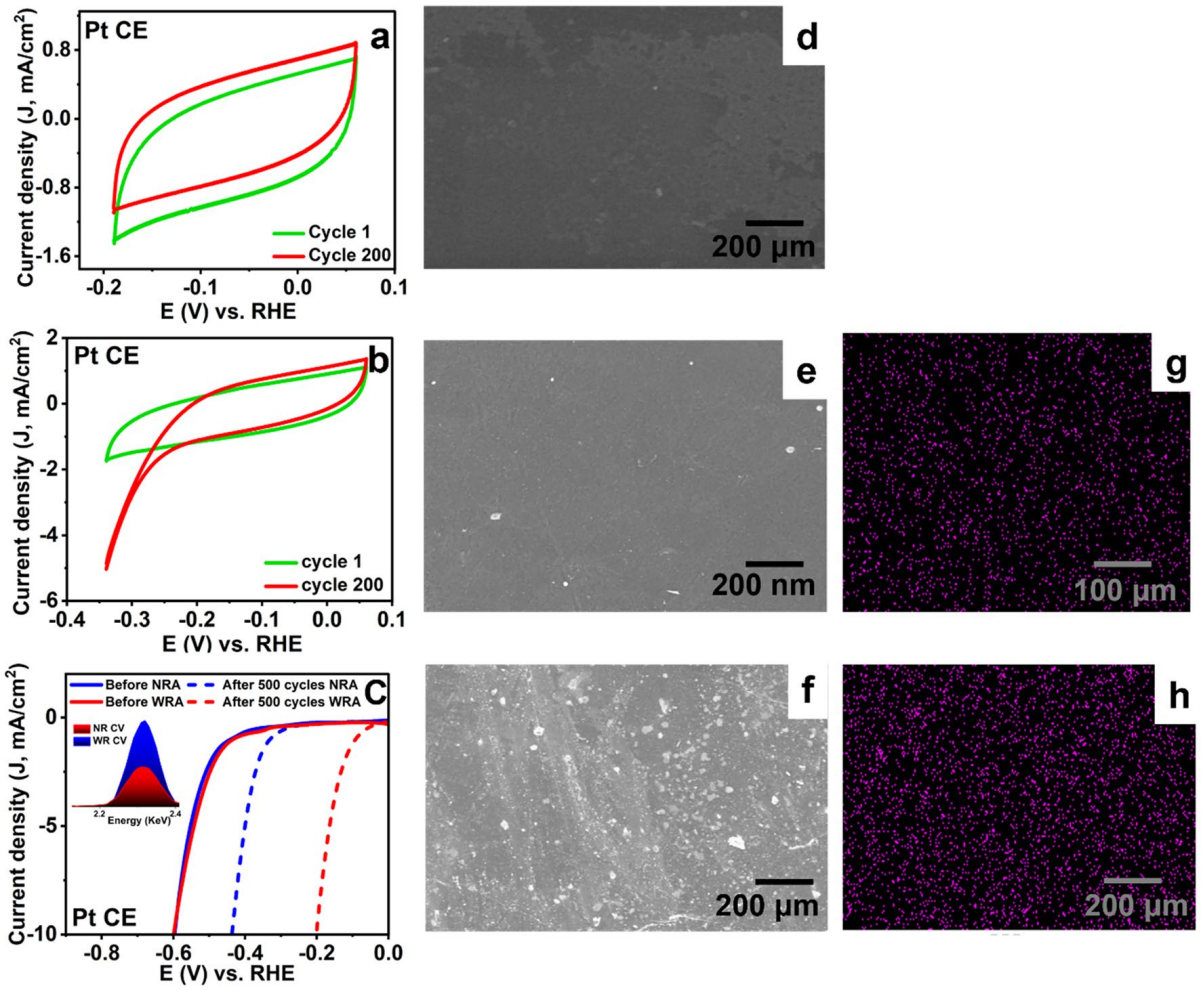
(**a**,**b**) CV scans using Pt foil as the CE upon varying the potential range, (c) LSV scans before and after applying the different potential ranges, the inset in (**c**) depicts the difference in the Pt EDX signal intensity after applying the different potential ranges. FESEM images of the sample (**d**) before and after applying (**e**) narrow and (**f**) wide activation range. (**g**,**h**) EDX mapping of the images shown in (**e**) and (**f**).

In most of the recently reported studies related to HER, activation or surface cleaning is the first step performed in electrochemical measurements. It is widely accepted that as the current increases, the potential on the counter electrode increases^4^. This encouraged us to investigate the effect of different potential windows during the activation step and its correlation to the counter electrode dissolution rate. Thus, we explored two ranges for the CV cycles; narrow activation range, NRA, (0.06 V to − 0.2 vs RHE) and wide activation range, WRA, (0.06 V to − 0.34 V vs RHE). Upon expanding the potential window towards more negative potentials, the current starts to increase as the number of cycles increases, Fig. 1a,b. This is expected to increase the rate of Pt dissolution. The dissolved Pt would redeposit on the surface of the working electrode, resulting in a deceptive enhancement in the recorded catalytic activity of the working electrode. Figure 1c shows the shift in the linear sweep voltammograms (LSV) recorded before and after the electrochemical activation at − 10 mA/cm^2^. When a narrow CV range scan is applied in the non-Faradaic region, a shift of 170 mV was recorded after 300 CV cycles, while a shift of 400 mV was observed after 300 CV cycles in the wide activation range. The EDX analysis of both samples after electrochemical measurements (inset in Fig. 1c) showed much higher intensity of the Pt peak of the sample tested in the wide activation range than that tested in the narrow range. Furthermore, the SEM images of the sample before electrochemical activation (Fig. 1d) and after applying narrow (Fig. 1e) and wide range (Fig. 1f) activation were investigated. Note the presence of a few number of small Pt nanoparticles upon applying narrow activation range. However, upon activation over a wide range, the surface was heavily covered with Pt nanoparticles. The corresponding EDX mappings of the surface of the working electrode after applying NRA and WRA are shown in Fig. 1g,h, respectively. This signifies the importance of choosing the activation range that does not involve any current increase to avoid the dissolution of the CE. Note that electrode polarization usually takes place upon attaining high current or if the electron transport rate is higher than the reaction rate^4^.

### Effect of the area of the working electrode on CE dissolution rate

**Figure 2:**
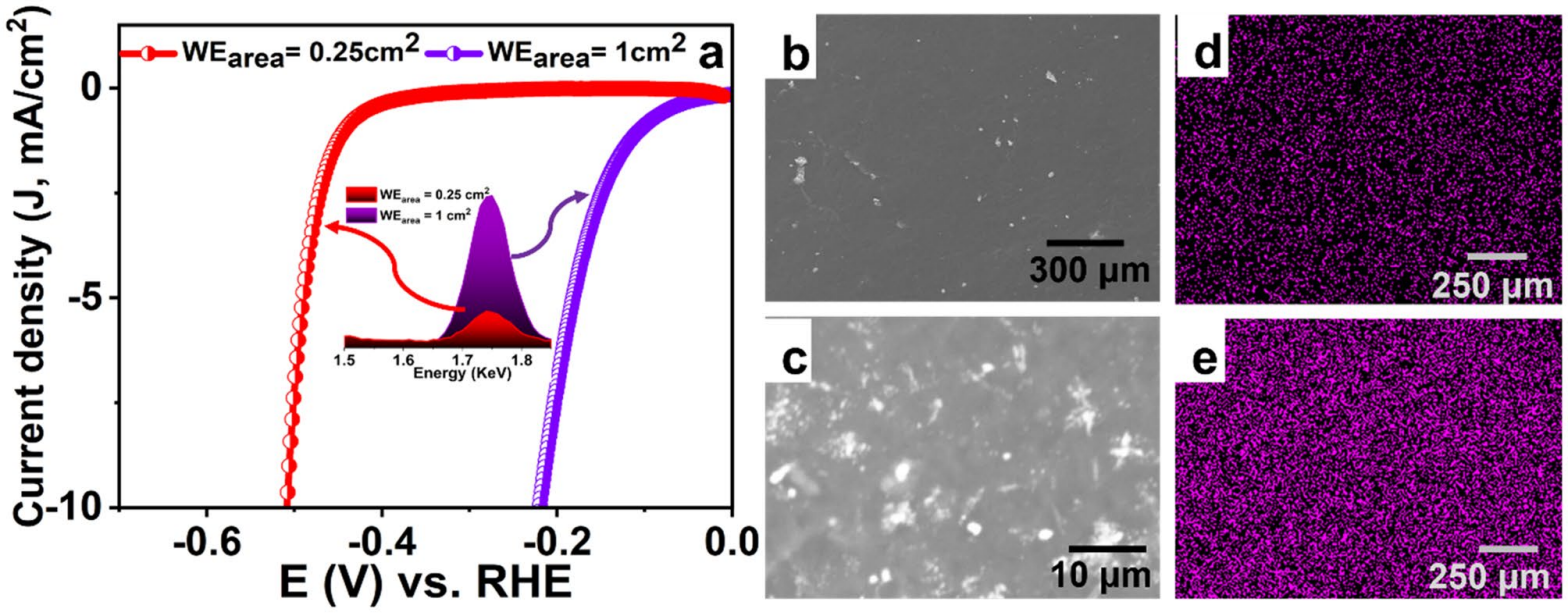
(**a**) LSV curves using Pt foil as the CE while varying the area of the WE, the inset in (**a**) shows the difference in EDX signal intensity of Pt deposited on of the two WEs after applying the electrochemical measurements, and (**b**,**c**) FESEM images of the WEs with the area of 0.25 cm^2^ and 1 cm^2^ after applying the electrochemical measurements, respectively.

Counter electrodes in electrochemical cells are used to keep the rate of the half-reaction taking place on their surfaces faster than the other half-reaction on the WE. Consequently, if the CE’s half-reaction is slower than the complementary one on the WE, the recorded current would be misleading as the CE dictates the current response. To overcome such problem, the CE should possess high electrical conductivity and larger surface area (typically ten times) than the WE to ensure fast reaction kinetics on the CE^9^. To elucidate such an impact, the area of the Pt CE was kept constant while varying the area of the WE (0.25 cm^2^ and 1 cm^2^). As the area of the WE increases, the overpotential drastically reduced (Fig. 2a), which can be ascribed to the higher rate of Pt dissolution and redeposition on the working electrode. This was further confirmed by the EDX analysis, inset in Fig. 2a, where the intensity of the Pt peak significantly increases as the area increases. Additionally, SEM images further confirm the correlation between the area of the working electrode and the dissolution rate of the CE, Fig. 2b,c. When the area of the WE was 0.25 cm^2^, small Pt nanoparticles start to appear on the surface of the WE. On the other hand, when the area of the WE was 1 cm^2^, highly dense Pt particles were observed on the WE surface, starting to form Pt clusters. The corresponding EDX mapping of the surface of the working electrode after electrochemical measurements are shown in Fig. 2d,e, respectively.

### Is there a way out?

**Figure 3:**
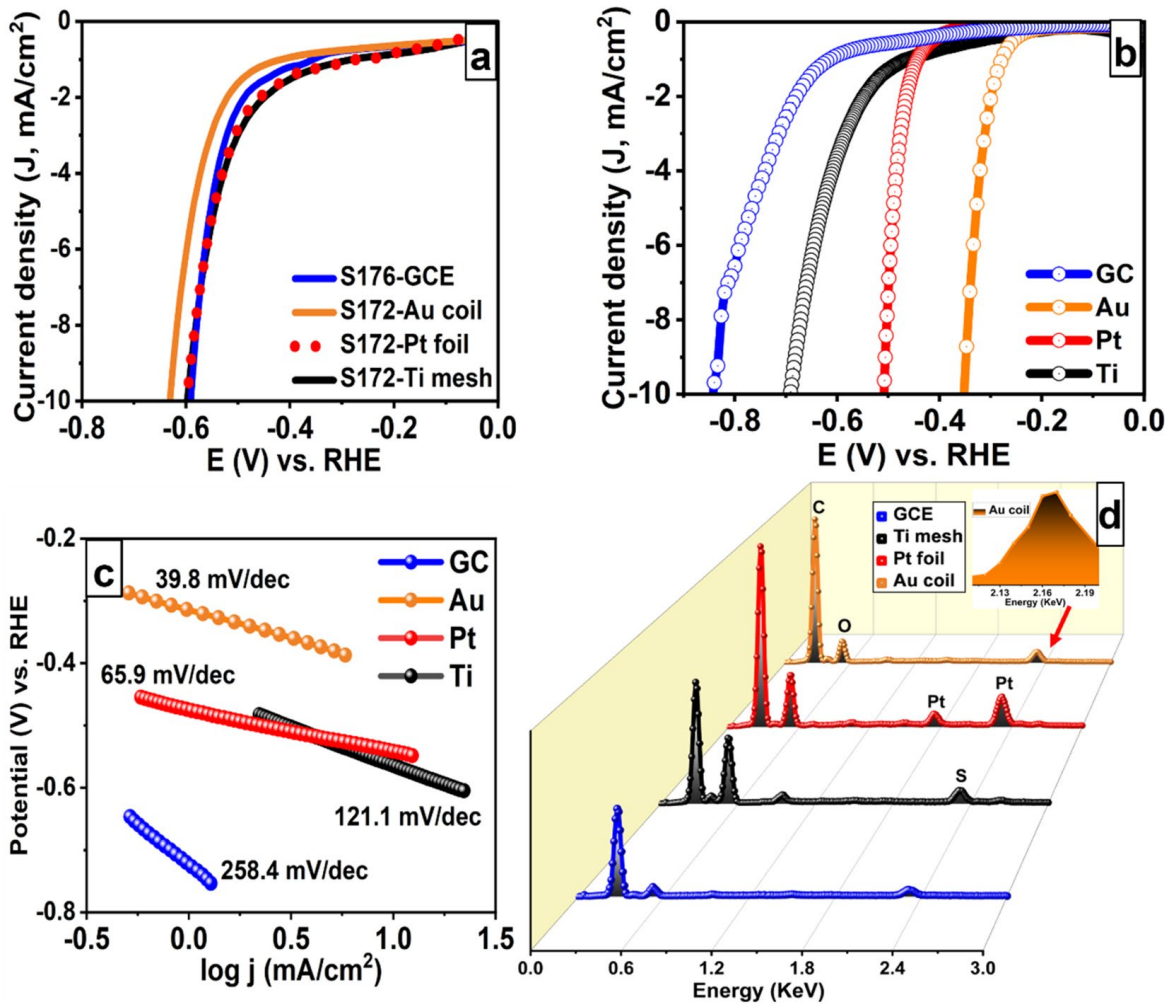
LSV scans (**a**) before and (**b**) after activation, (**c**) the corresponding Tafel slopes, and (**d**) EDX of the working electrodes after electrochemical measurements using Pt foil, gold coil, glassy carbon rod, and Ti mesh roll as the counter electrodes.

The above findings showed the dissolution of Pt upon its use as a CE for HER in acidic media. Therefore, more materials have been tested to elucidate their stability with the hope to identify a stable material for use as CE. Particularly, Pt foil, gold coil, glassy carbon rod, and titanium mesh roll were tested as the counter electrodes in HER 3-electrode system. LSV experiments were performed while fixing the area of the working electrode at 0.25 cm^2^. The overpotentials were measured for the different counter electrodes before and after applying 700 activation cycles in the narrow activation range (0.06 V to − 0.2 V vs RHE) at a scan rate of 5 mVs^−1^ in 0.5 M H_2_SO_4_. Figure 3a shows the LSV scans of the four counter electrodes before activation, revealing almost the same overpotential for the four counter electrodes. Figure 3b shows the LSV scans of the four counter electrodes after activation Note that there is a substantial shift in the recorded overpotential. The recorded overpotentials are in ascending order as gold coil, Pt foil, titanium mesh, and glassy carbon rod, with the values of − 0.35 V, − 0.5, − 0.69, and − 0.84 V, respectively. While the lowest overpotential was recorded upon the use of gold coil as the CE, the use of glassy carbon electrode resulted in the highest overpotential. Tafel slopes were calculated to investigate the kinetics of the HER^10^, as shown in Fig. 3c. When gold coil was used as the CE, the lowest Tafel slope was obtained (39.8), while the Tafel slope of the Pt, Ti mesh, and glassy carbon rod CE were 65.9, 121.1, and 258.4, respectively. The enhancement in the overpotential and the Tafel slope values when gold coil and Pt foil were used as the CEs indicates that this is probably due to the dissolution and redeposition of gold and platinum on the working electrode. We expect the enhancement in the reaction kinetics is due to the deposition of gold and Pt species on the surface of the working electrode, resulting in reduced overpotential, in accordance with the LSV measurements discussed before. Besides, the shift observed after activation suggests that electrochemical dissolution mainly happens during the activation cycles. The EDX analysis (Fig. 3d) revealed the presence of gold and platinum on the working electrode, while no Ti was detected.

**Figure 4:**
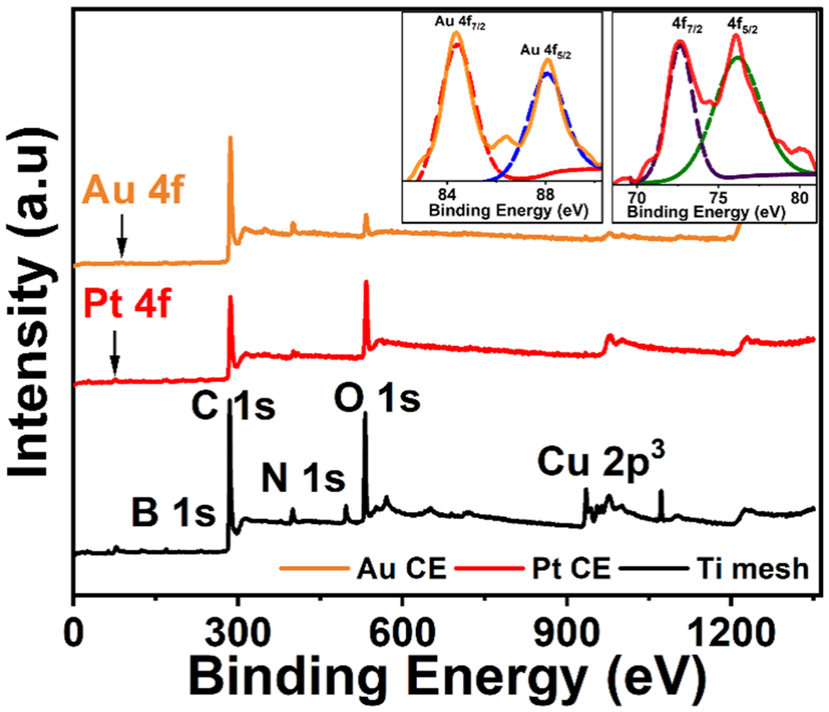
XPS survey of the working electrode after electrochemical measurements using Pt foil, Au coil, and Ti mesh as the counter electrode, the inset is for Pt and Au 4f spectrum.

Moreover, XPS analysis further confirmed the deposition of Pt and Au nanoparticles on the surface of the counter electrodes, as illustrated in Fig. 4, upon the use of Pt foil and Au coil as the counter electrodes. The spin–orbit coupling of Au 4f_7/2_ and Au 4f_5/2_ is 84 eV and 87.5 eV, indicating the presence of elemental Au^0^ on the surface of the working electrode^11,12^. Also, Pt 4f_7/2_ and Pt 4f_5/2_ peaks were observed at 71.6 eV and 74.9 eV respectively^4,13,14^. On the other hand, upon using Ti mesh as the CE, no signal related to Ti was detected. This indicates that both gold coil and platinum foil are not stable. On the other hand, although Ti mesh and glassy carbon rod are stable, a large shift in the overpotential is evident, which is probably due to the small surface area of the glassy carbon rod relative to the large surface area of the Ti mesh roll. Yi et al*.* demonstrated that glassy carbon in acidic media undergoes degradation because the acid catalyses the formation of surface oxides followed by ring opening in the graphitic structure, and finally oxidation in the bulk. Thus, glassy carbon rod does not seem to be efficient in elucidating the catalytic activity of the working electrode and it might result in misleading overpotential^15^.

## Conclusion

In conclusion, the effect of the area of the working electrode relative to the area of counter electrode on the performance of the catalyst for HER in acidic media was demonstrated. Choosing the convenient potential range for the activation/cleaning of the surface is very crucial as it might accelerate the dissolution rate of the counter electrode. To this end, the dissolution behaviour of four different counter electrodes was investigated during the electrochemical measurement of HER activity in acidic medium. Gold coil, Pt foil, and glassy carbon counter electrodes were found to be susceptible to dissolution in acidic medium. The dissolved ions were redeposited on the surface of the working electrode, resulting in fictitious enhancement in the recorded overpotential and the electrochemical activity of the working electrode. On the good side, the Ti mesh, when used as the counter electrode, showed exceptional stability with no Ti detected on the working electrode surface as confirmed via EDX and SEM analyses.

## Data Availability

The datasets used and/or analysed during the current study available from the corresponding author on reasonable request.
